# Comparative anatomical outcomes of high-flow vs. low-flow phacoemulsification cataract surgery: A systematic review and meta-analysis

**DOI:** 10.3389/fmed.2022.1021941

**Published:** 2022-09-28

**Authors:** Po-Chin Kuo, Jia-Horung Hung, Yu-Chen Su, Ching-Ju Fang, Chaw-Ning Lee, Yi-Hsun Huang, Shih-Chieh Shao, Edward Chia-Cheng Lai

**Affiliations:** ^1^Education Center, National Cheng Kung University Hospital, College of Medicine, National Cheng Kung University, Tainan, Taiwan; ^2^Department of Ophthalmology, National Cheng Kung University Hospital, College of Medicine, National Cheng Kung University, Tainan, Taiwan; ^3^Institute of Clinical Medicine, College of Medicine, National Cheng Kung University, Tainan, Taiwan; ^4^Medical Library, National Cheng Kung University, Tainan, Taiwan; ^5^Department of Secretariat, National Cheng Kung University Hospital, College of Medicine, National Cheng Kung University, Tainan, Taiwan; ^6^Department of Dermatology, National Cheng Kung University Hospital, College of Medicine, National Cheng Kung University, Tainan, Taiwan; ^7^School of Pharmacy, Institute of Clinical Pharmacy and Pharmaceutical Sciences, College of Medicine, National Cheng Kung University, Tainan, Taiwan; ^8^Department of Pharmacy, Keelung Chang Gung Memorial Hospital, Keelung, Taiwan

**Keywords:** phacoemulsification, cataract surgery, fluidics, endothelial cell loss, central corneal thickness, central macular thickness, systematic review, meta-analysis

## Abstract

**Background:**

Phacoemulsification is an effective and widely performed technique in cataract surgery, but the comparative anatomical outcomes, including endothelial cell loss (ECL), central corneal thickness (CCT), and central macular thickness (CMT), between high-flow and low-flow phacoemulsification cataract surgery remain unclear.

**Methods:**

This study followed Preferred Reporting Items for Systematic Reviews and Meta-Analyses (PRISMA) Statement. Random-effects models were applied to measure pooled mean differences (MD) with 95% confidence intervals (CI) of anatomical outcomes between high-flow and low-flow phacoemulsification cataract surgery. We judged overall certainty of evidence (CoE) based on Grading of Recommendations Assessment, Development and Evaluation (GRADE) criteria.

**Results:**

We included six randomized controlled trials (RCTs) totaling 477 participants. The meta-analysis showed similar changes associated with these two surgery types in both ECL at postoperative days 2–14 (MD: −1.63%; 95% CI: −3.73 to 0.47%; CoE: very low), days 15–42 (MD: −0.65%; 95% CI −2.96 to 1.65%; CoE: very low) and day 43 to month 18 (MD: −0.35%; 95% CI: −1.48 to 0.78%; CoE: very low), and CCT at postoperative day 1 (MD: −16.37 μm; 95% CI: −56.91 to 24.17 μm; CoE: very low), days 2–14 (MD: −10.92 μm; 95% CI: −30.00 to 8.16 μm; CoE: very low) and days 15–42 (MD: −2.76 μm; 95% CI: −5.75 to 0.24 μm; CoE: low). By contrast, low-flow phacoemulsification showed less increase in CMT at postoperative days 15–42 (MD, −4.58 μm; 95% CI: −6.3 to −2.86 μm; CoE: low).

**Conclusions:**

We found similar anatomical outcomes, except in CMT, for both high-flow and low-flow phacoemulsification cataract surgery. Future head-to-head RCTs on visual outcomes should confirm our findings.

**Systematic review registration:**

PROSPERO, identifier: CRD42022297036.

## Introduction

Senile cataract, as the leading cause of blindness and the second most common cause of moderate and severe vision impairment according to the Global Burden of Disease, has a prevalence estimated at around 54.38% among populations over 60 years old (1). Visual impairment caused by cataract can be restored through timely cataract surgery. Phacoemulsification is an effective and widely performed technique for cataract surgery, with estimates exceeding 11,000 surgeries/million population in the US in 2011 (2). During phacoemulsification cataract surgery, irrigation fluid circulates through the eye intraoperatively to both maintain intraocular pressure and provide cooling in order to prevent ocular tissue damage while the lens nucleus is being emulsified through the use of ultrasound energy.

However, the optimal flow rate for phacoemulsification cataract surgery is not yet determined. High-flow settings, with aspiration flow rates ranging from 35 to 50 ml/min (3, 4) are generally considered to have higher efficiency. They are preferred by many surgeons for rock-hard dense cataract cases to improve the vacuum purchase of the hard nuclei, and to decrease phaco tip clogging (5), and also create sufficient space in the anterior segment for surgical manipulation, at the expense of risking surgical trauma caused by turbulence (3). Low-flow settings, often with aspiration flow rates lower than 25 ml/min (3), prioritize safety because they create a more stable intraoperative environment, theoretically decreasing the chance of intraoperative complications such as posterior capsule rupture. They are therefore as well recommended when dealing with cases with zonular insufficiency (6). However, they may require a longer surgical time, and limit surgeons to a relatively small space in the anterior segment (7). In clinical practice, ophthalmologists decide the fluidics settings based only on their own surgical experience.

Phacoemulsification by ultrasound energy is known to cause corneal endothelial cell loss (ECL) (8). In addition, phacoemulsification may also trigger inflammation, thus leading to macular edema. However, several randomized controlled trials (RCTs) with small sample sizes reported inconsistent surgical outcomes, regarding ECL, central corneal thickness (CCT) and central macular thickness (CMT), after high- or low-flow phacoemulsification cataract surgery (4, 9–13). To summarize the current evidence and better inform clinical decision-making, we conducted a systematic review and meta-analysis study on the surgical outcomes reported from RCTs comparing high-flow and low-flow phacoemulsification cataract surgery.

## Methods

This systematic review and meta-analysis followed the Preferred Reporting Items for Systematic Reviews and Meta-Analyses (PRISMA) guidelines (14). The review protocol was registered on PROSPERO (CRD42022297036) prior to conducting the review.

### Literature search

We searched for published RCTs *via* Medline, Embase, Cochrane Central Register of Controlled Trials and Scopus on July 8, 2022. The literature search was limited to human studies, with no language restrictions. The search strategy was developed by an experienced librarian (CJF), and the details are presented in Supplementary Table S1. To capture any unpublished studies, we also consulted the pharmaceutical and medical device companies associated with these cataract surgeries for additional studies.

### Study eligibility

We included RCTs comparing high-flow and low-flow fluidic settings for phacoemulsification. We recruited studies with patients randomly assigned to either high-flow or low-flow phacoemulsification. Fluidic settings of the phacoemulsification surgery were to be clearly stated.

### Study selection

Two review authors (PCK and JHH) independently selected eligible literatures based on the pre-specified inclusion criteria, including (1) Participants: cataract patients; (2) Intervention/Comparison: high-flow or low-flow phacoemulsification; (3) Primary study outcome: endothelial cell loss; (4) Study design: RCTs. We initially screened records by titles and abstracts to identify potential candidates, and then the review authors reviewed their full texts to select those for inclusion. Any discrepancy between the review authors was resolved by discussion with the third review author (SCS) before final decision.

### Data extraction

Two review authors (PCK and JHH) independently extracted data including first author, publication year, country, study design, patient characteristics, interventions and comparators (surgical modes and parameter settings), and outcome measures from the included studies. In addition to the primary anatomic outcome of endothelial cell loss (ECL), we extracted other important anatomic parameters as secondary outcomes, including central corneal thickness (CCT) and central macular thickness (CMT). We investigated CCT because postoperative corneal thickness is known as a marker of endothelial damage after phacoemulsification (15). We also investigated CMT because phacoemulsification surgery commonly leads to postoperative macular edema (16), which may affect postoperative visual outcomes. Any discrepancy between the review authors was resolved by discussion.

### Risk of bias

Two review authors (PCK and JHH) independently assessed the risk of bias in the included studies with the Cochrane Risk of Bias tool 1.0 (17). The domains, including random sequence generation, allocation concealment, blinding of participants and personnel, blinding of outcome assessment, incomplete outcome data, selective reporting, and other biases, were categorized as high-, low- or unclear risk of bias. If an RCT did not report data of adverse effects, we rated it as having a high risk of selective reporting bias. We also judged there to be a high risk of other bias if a baseline imbalance was found between the intervention- and control groups after randomization. Any discrepancy between the review authors was resolved by discussion with the third review author (SCS) before final decision.

### Data synthesis

We conducted all meta-analyses using Review Manager version 5.3.4.1 (Copenhagen: The Nordic Cochrane Center, The Cochrane Collaboration, 2020). Random-effects models were applied to measure the pooled mean differences (MD) and 95% confidence intervals (CI) of the study outcomes of interest comparing high-flow and low-flow phacoemulsification cataract surgery. We analyzed the study outcomes based on four follow-up time periods to evaluate the immediate (i.e., postoperative day 1), short-term (i.e., postoperative days 2–14), intermediate-term (i.e., postoperative days 15–42), and long-term (i.e., postoperative day 43 to month 18) comparative treatment effects between high-flow and low-flow phacoemulsification cataract surgery.

To achieve concordance between included studies, extracted outcome data from included studies were adjusted using reasonable statistical methods. For example, ECL was calculated as percentage change based on the formula: (postoperative ECL – preoperative ECL)/(preoperative ECL), while CCT and CMT were calculated as μm change based on the formula: (postoperative value – preoperative value). ECL was calculated as percentage change because absolute value data were not available in the studies from Baradaran-Rafii 2009 (11) and Schriefl 2014 (4). Where only the median and range of outcome measures were available, we estimated the mean and variance through the specific formula (18). We assessed statistical heterogeneity among included studies by *I*^2^ statistic, and considerable heterogeneity was defined as an *I*^2^ >50% (17). Publication bias was to be evaluated by funnel plots if over 10 studies were included for meta-analysis (17).

### Overall certainty of evidence

Two independent reviewers (PCK and JHH) assessed the overall certainty of evidence (CoE) for each study outcome based on the Grading of Recommendations Assessment, Development and Evaluation (GRADE) criteria (19). Any discrepancy between the review authors was resolved by discussion with the third review author (SCS) before final decision.

## Results

### Characteristics of included studies

The study selection flowchart is presented in Supplementary Figure S1. We initially identified a total of 1,111 records through the systematic search, and after screening the study titles and abstracts, 12 potential articles (4, 9–13, 20–25) were evaluated for final eligibility. Of these potential records, we excluded two studies which were not designed as randomized controlled trials (23, 24), one study lacking comparisons between high-flow and low-flow phacoemulsification cataract surgery (25), and three studies without final reports (20–22). Ultimately, in this systematic review and meta-analysis, we only included six reports (4, 9–13), with a total of 477 participants from six RCTs.

Table 1 summarizes the study, participant and surgery characteristics, outcomes, and main findings of the included RCTs. These RCTs recruited participants from Iran, Sweden, India and Austria. Briefly, all RCTs included participants undergoing phacoemulsification for senile cataract with the mean age ranging from 53 to 74 years old. In two RCTs (12, 13) phacoemulsification was performed by longitudinal ultrasound, in two RCTs (9, 10) it was by torsional ultrasound, in one RCT (11) by transversal ultrasound, and the last RCT (4) did not specify the mode of ultrasound used. The flow rate ranged from 35 to 45 cc/min in the high-flow group, and from 20 to 25 cc/min in the low-flow group. The vacuum pressure ranged from 400 to 650 mmHg in the high-flow group, and from 200 to 400 mmHg in the low-flow group.

**Table 1:** Characteristics of comparative studies regarding high-low and low-flow aspiration flow settings of patients who underwent phacoemulsification.

**Study**	**Country**	**Population**	**Intervention and comparator**	**Outcomes**	**Main findings**
Baradaran-Rafii et al. (2009) (11)	Iran	50 to 70-year-old, senile cataract with 3+ nuclear sclerosis Mean age: High-flow (*n* = 30) 61.4 ± 4.9 Low-flow (*n* = 30) 60.8 ± 6.6	WhiteStar^®^, Transversal ultrasound High-flow (400 mmHg, 40 cc/min) Low-flow (200 mmHg, 20 cc/min)	ECL (postoperative week 1, 6, 12)	ECL: low-flow 9.5%, high-flow 10.6% at week 1 (*p* = 0.6); low-flow 8.7%, high-flow 9.1% at week 6 (*p* = 0.8); low-flow 9.6%, high-flow 9.0% at week 12 (*p* = 0.6)
Chang et al. (2017) (9)	Sweden	50 to 85-year-old, senile cataract Mean age: High-flow (*n* = 21) 68.5 ± 8 Low-flow (*n* = 22) 70.5 ± 8.6	Infiniti^®^, Torsional ultrasound High-flow (475 mmHg, 45cc/min) Low-flow (350 mmHg, 22cc/min)	1. ECL (postoperative month 3) 2. CCT (postoperative day 1, week 3, month 3) 3. CMT (postoperative day 1, week 3, month 3)	1. ECL: low-flow 194 cells/mm^2^, high-flow 279 cells/mm^2^ at month 3 (*p* = 0.46) 2. CCT change: low-flow 35 μm, high-flow 27 μm at day 1 (*p* = 0.51); low-flow 9 μm, high-flow 17.5 μm at week 3 (*p* = 0.48); low-flow 1 μm, high-flow 4 μm at month 3 (*p* = 0.91) 3. CMT change: low-flow −1.5 μm, high-flow 0 μm at day 1 (*p* = 0.57); low-flow 11.5 μm, high-flow 16 μm at week 3 (*p* = 0.39); low-flow 10 μm, high-flow 13.5 μm at month 3 (*p* = 0.91)
Das et al. (2015) (10)	India	Senile cataract (LOCS III grade 2–4) Mean age: High-flow (*n* = 65) 64.9 ± 9.19 Low-flow (*n* = 65) 63.94 ± 7.84	Infiniti^®^, Torsional ultrasound High-flow (450–500 mmHg, 40–45 cc/min) Low-flow (300–350 mmHg, 25cc/min)	1. ECL (postoperative week 2, 6) 2. CCT (postoperative week 2, 6) 3. CMT (postoperative week 6)	1. ECL: low-flow 245.82 cells/mm^2^, high-flow 320.70 cells/mm^2^ at week 2 (*p* = 0.997); low-flow 243.24 cells/mm^2^, high-flow 282.93 cells/mm^2^ at week 6 (*p* = 0.135) 2. CCT change: low-flow 0.24 μm, high-flow 1.41 μm at week 2 (*p* = 0.110); low-flow 1.76 μm, high-flow 3.41 μm at week 6 (*p* = 0.197) 3. CMT change: low-flow 0 μm, high-flow 3.22 μm at week 6 (*p* = 0.393)
Schriefl et al. (2014) (4)	Austria	Senile cataract Mean age: 74 ± 9 High-flow (*n* = 57) Low-flow (*n* = 57)	OS3 base module^®^, High-flow (500 mmHg, 35 cc/min) Low-flow (400 mmHg, 20 cc/min)	ECL (postoperative week 1, month 18)	ECL: low-flow 1.8%, high-flow 4.46% at week 1 (*p* = 0.449); low-flow 4.92%, high-flow 6.26% at month 18 (*p* = 0.696)
Vasavada et al. (2010) (12)	India	Senile cataract (Emery-Little classification grade 2–3) Mean age: High-flow (*n* = 25) 53 ± 2.7 Low-flow (*n* = 25) 59 ± 3.1	Infiniti^®^, Longitudinal ultrasound High-flow (≤650 mmHg, 40 cc/min) Low-flow (≤400 mmHg, 25 cc/min)	1. ECL (postoperative month 3) 2. CCT (postoperative day1, week 1, month 1, 3)	1. ECL: low-flow 4.67%, high-flow 5.22% at month 3 (*p* = 0.45) 2. CCT change: low-flow 6.49%, high-flow 13.44% at day 1 (*p* < 0.001); low-flow 1.74%, high-flow 5.55% at week 1 (*p* < 0.001); low-flow 1.49%, high-flow 1.86% at month 1 (*p* = 0.2); low-flow 0.79%, high-flow 1.11% at month 3 (*p* = 0.14)
Vasavada et al. (2014) (13)	India	Senile cataract, LOCS III grade 2–3 Mean age: High-flow (*n* = 40) 62.67 ± 8.79 Low-flow (*n* = 40) 64.42 ± 5.43	Infiniti^®^, Longitudinal ultrasound High-flow (400 mmHg, 40 cc/min) Low-flow (400 mmHg, 20 cc/min)	1. ECL (postoperative month 3) 2. CCT (postoperative day 1, week 1, month 1)	1. ECL: No statistically significant percentage change at month 3 (no exact value provided) 2. CCT change: low-flow 6.42%, high-flow 13.28% at day 1 (*p* < 0.001); low-flow 1.71%, high-flow 5.51% at week 1 (*p* < 0.001); low-flow 1.47%, high-flow 1.86% at month 1 (*p* = 0.2)

ECL, endothelial cell loss; CCT, central corneal thickness; CMT, central macular thickness; LOCS, lens opacities classification system.

### Risk of bias

The overall risk of bias assessment is presented in Supplementary Figure S2, and the authors' detailed judgements for each domain of the risk of bias tools are presented in Supplementary Table S2. We considered most of the included RCTs (5/6) to have performance bias, since the surgeons were not blinded to the intervention (4, 9, 10, 12, 13). In addition, we found 5 RCTs may have suffered from selection bias (4, 9–12) and detection bias (4, 9, 11). Finally, we judged two RCTs as having other bias (12, 13), because, despite having a similar study population source but different sample sizes and surgical parameters, they reported exactly the same outcome data for preoperative and postoperative CCT.

### Primary anatomic outcome: ECL

**Figure 1:**
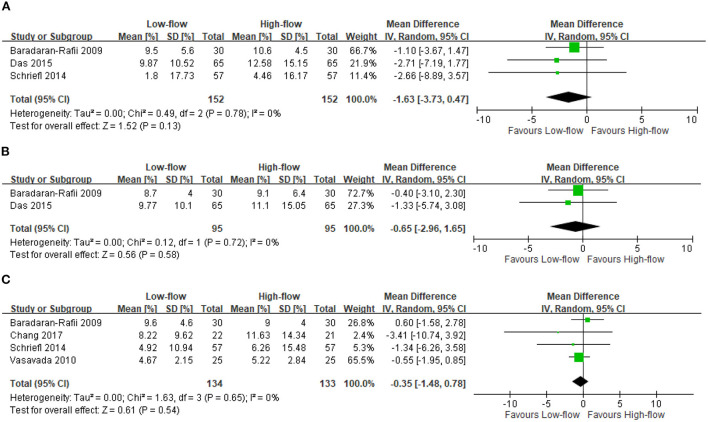
Endothelial cell loss (ECL). **(A)** Postoperative days 2–14 [%]. **(B)** Postoperative days 15–42 [%]. **(C)** Postoperative day 43 - month 18 [%].

We found five RCTs reporting the ECL changes after high-flow or low-flow phacoemulsification cataract surgery (4, 9–12). The meta-analysis showed no significant differences in ECL at postoperative days 2–14 (three RCTs; 304 participants; MD: −1.63%; 95% CI: −3.73 to 0.47%; *I*^2^ = 0%) (4, 10, 11) (Figure 1A), at days 15–42 (2 RCTs; 190 participants; MD: −0.65%; 95% CI: −2.96 to 1.65%; *I*^2^ = 0%) (10, 11) (Figure 1B), and at day 43 to month 18 (four RCTs; 267 participants; MD: −0.35%; 95% CI: −1.48 to 0.78%; *I*^2^ = 0%) (4, 9, 11, 12) (Figure 1C). However, the included RCTs lacked data regarding the differences in ECL between these two surgery types at postoperative day 1.

### Secondary anatomic outcomes: CCT, CMT

**Figure 2:**
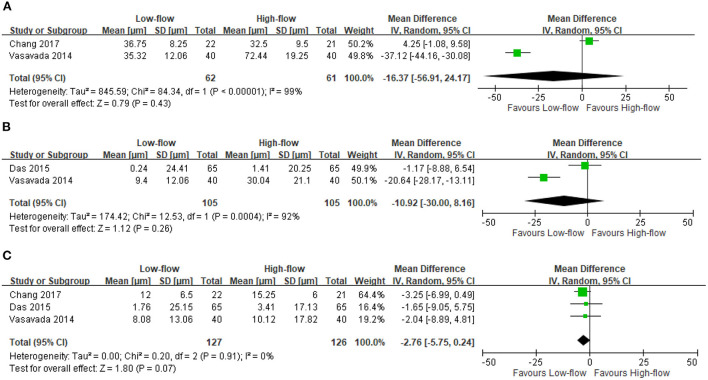
Change in central corneal thickness (CCT). **(A)** Postoperative day 1 [μm]. **(B)** Postoperative days 2–14 [μm]. **(C)** Postoperative days 15–42 [μm].

We found three RCTs reporting the CCT changes after high-flow or low-flow phacoemulsification cataract surgery (9, 10, 13). The meta-analysis showed no significant differences in CCT changes at postoperative day 1 (two RCTs; 123 participants; MD: −16.37 μm; 95% CI: −56.91 to 24.17 μm; *I*^2^ = 99%) (9, 13) (Figure 2A), at days 2–14 (two RCTs; 210 participants; MD: −10.92 μm; 95% CI: −30 to 8.16 μm; *I*^2^ = 92%) (10, 13) (Figure 2B) and at days 15–42 (three RCTs; 253 participants; MD: −2.76 μm; 95% CI: −5.75 to 0.24 μm; *I*^2^ = 0%) (9, 10, 13) (Figure 2C). However, the included RCTs lacked data regarding the differences in CCT changes between these two surgery types at postoperative day 43 to month 18.

**Figure 3:**
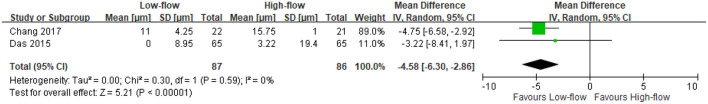
Change in central macular thickness (CMT), postoperative days 15–42 [μm].

We found two RCTs reporting the CMT changes after high-flow or low-flow phacoemulsification cataract surgery (9, 10). The meta-analysis showed less increase in CMT after low-flow phacoemulsification at postoperative days 15–42 (two RCTs; 173 participants; MD: −4.58 μm; 95% CI: −6.3 to −2.86 μm; *I*^2^ = 0%) (9, 10) (Figure 3). However, the included RCTs lacked data regarding the differences in CMT changes between these two surgery types at postoperative days 1–14, and day 43 to month 18.

### Overall certainty of evidence

Table 2 summarizes the main findings and certainty of evidence for each pooled outcome estimate. All outcome measures were downgraded due to risk of bias and imprecision. The overall certainty of evidence ranged from very low to low.

**Table 2:** Summary of anatomical outcomes from low-flow compared with high-flow phacoemulsification.

	**Quality assessment**					**Summary of findings**	
**Participants (studies)**	**Risk of bias**	**Inconsistency**	**Indirectness**	**Imprecision**	**Publication bias**	**Mean difference (95% CI)**	**Overall certainty of evidence (CoE)**
**Endothelial cell loss (ECL)**							
Postoperative day 1							
No RCTs	NA	NA	NA	NA	NA	NA	NA
Postoperative days 2–14							
304 (3 RCTs)	Very serious	Not serious	Not serious	Serious	Not detected	−1.63 % (−3.73 to 0.47)	⊕○○○ Very low^*^^‡^
Postoperative days 15–42							
190 (2 RCTs)	Very serious	Not serious	Not serious	Serious	Not detected	−0.65 % (−2.96 to 1.65)	⊕○○○ Very low^*^^‡^
Postoperative day 43 to month 18							
267 (4 RCTs)	Very serious	Not serious	Not serious	Serious	Not detected	−0.35 % (−1.48 to 0.78)	⊕○○○ Very low^*^^‡^
**Change in central corneal thickness (CCT)**							
Postoperative day 1							
123 (2 RCTs)	Very serious	Serious	Not serious	Serious	Not detected	−16.37 μm (−56.91 to 24.17)	⊕○○○ Very low^*^^†^^‡^
Postoperative days 2–14							
210 (2 RCTs)	Very serious	Serious	Not serious	Serious	Not detected	−10.92 μm (−30 to 8.16)	⊕○○○ Very low^*^^†^^‡^
Postoperative days 15–42							
253 (3 RCTs)	Serious	Not serious	Not serious	Serious	Not detected	−2.76 μm (−5.75 to 0.24)	⊕⊕○○ Low^*^^‡^
Postoperative day 43 to month 18							
No RCTs	NA	NA	NA	NA	NA	NA	NA
**Change in central macular thickness (CMT)**							
Postoperative days 1–14							
No RCTs	NA	NA	NA	NA	NA	NA	NA
Postoperative days 15–42							
173 (2 RCTs)	Serious	Not serious	Not serious	Serious	Not detected	−4.58 μm (−6.3 to −2.86)	⊕⊕○○ Low ^*^^‡^
Postoperative day 43 to month 18							
No RCTs	NA	NA	NA	NA	NA	NA	NA

* Risk of bias: no concerns (≥75% cells with low or no risk of bias and no cell with high risk of bias), serious concerns (no cell with high risk of bias and >25% cells with unclear risk of bias, or at least one but <25% cells with high risk of bias and ≤ 25% cells with unclear risk of bias), and very serious concerns (≥25% cells with high risk of bias, or at least one cell with high risk of bias and ≥25% cells with unclear risk of bias).

† Inconsistency: I^2^ >50%.

‡ Imprecision: total study participants < 400.

## Discussion

This systematic review and meta-analysis of 6 RCTs with 477 participants mostly with senile cataract found that, compared to low-flow phacoemulsification cataract surgery, high-flow phacoemulsification cataract surgery led to a greater increase in postoperative CMT, while no differences were observed in postoperative ECL and CCT. Since the overall CoE for these comparisons was judged as low to very low, whether the conclusions can be fully applied in clinical decisions is uncertain.

The results of our analysis showed that high-flow fluidic settings triggered greater increase in postoperative CMT. Pseudophakic cystoid macular edema (PCME), with a post-phacoemulsification incidence ranging from 0.1% to 2.35%, is one of the complications after cataract surgery, and may lead to long-term visual deterioration that is difficult to treat. We included an analysis of the impact of different fluidic parameters on postoperative CMT, since abundant research has demonstrated that cystoid macular edema may occur even after uncomplicated phacoemulsification procedures (26, 27). Possible reasons for this are as follows: Higher vacuum level is often set together with higher aspiration flow rate to achieve the intended efficiency (28). Consequently, high-flow fluidics are associated with postocclusion surge and thus increase risk of posterior capsule rupture due to anterior chamber shallowing (29). Postocclusion surge brought about by high-flow, high-vacuum fluidics leads to greater intraoperative maximum IOP and greater IOP fluctuation (13), which may induce oxidative stress and damage the blood-retinal barrier, subsequently giving rise to macular edema (30). Also, IOP fluctuations may carry the risk of unstable orbital blood flow and oxygen supply, causing oxidative stress and further resulting in cystoid macular edema after phacoemulsification (31). Although our study showed that low-flow fluidic settings resulted in less increase in postoperative CMT, it is not known whether this is clinically significant. We reviewed previous studies to help find correlations. Bamahfouz A. has reported that CMT changes correlate with best-corrected vision changes in the first month after phacoemulsification cataract surgery (32). Greater macular thickness is also reported to be related to worse mesopic visual acuity (33). This raised our concerns about a greater increase in postoperative CMT implying the development of PCME. By optimizing the fluidic settings, we hope we can reduce the risk of PCME, thus relieving the treatment burden for the elderly after cataract surgery.

Our results showed no differences in postoperative ECL and CCT between patients operated on with high-flow or low-flow fluidic settings. For the purpose of assessing postoperative outcomes regarding the corneal structure, previous studies have demonstrated the importance of documenting corneal thickness and endothelial cell status (34, 35); hence, our study investigated both postoperative ECL and CCT changes in the two groups under different fluidics settings. We hypothesized that the advantages of low-flow fluidic settings, namely less turbulence and less damage to corneal endothelial cells, may be offset by longer surgical time and higher cumulative dissipated energy (11). It is also possible that the effect on CCT change might be transient and reversible, based on clinical findings wherein corneal edema is usually noted on postoperative day 1 and gradually resolves over the course of weeks. Therefore, our result suggested that the selection of high-flow or low-flow fluidic settings not necessarily be based on preoperative corneal parameters, including endothelial cell density or CCT.

To evaluate the overall CoE of the study outcomes from the present meta-analysis, we have applied the GRADE system for ECL, CCT and CMT. We judged the CoE of these outcomes as low to very low, possibly because of the serious risk of bias or result inconsistency among included studies and the wide confidence intervals of pooled result estimates. First, most included RCTs were vulnerable to performance bias (i.e., non-blinded designs), whereby this is unavoidable with surgical interventions like phacoemulsification cataract surgery. Detection bias was another common source of bias in the included studies. Since our study outcomes, including ECL, CCT and CMT changes, were obtained as objective values from instrument examinations in ophthalmology clinics, we considered such detection bias may not have seriously affected the result estimates. Second, significant statistical heterogeneity was observed in the CCT comparisons at postoperative days 1–14. Christakis et al. and previous studies have reported that torsional ultrasound phacoemulsification, compared to other modes of ultrasound, caused less chatter and less corneal edema postoperatively (36, 37). Hence, given that different modes of phacoemulsification, such as longitudinal and torsional ultrasound, were used in the included RCTs, the effect sizes of these RCTs were inconsistent. Third, we found wide confidence intervals around the ECL, CCT and CMT outcomes because relatively few patients were included in the meta-analysis. To reach more definite conclusions about the comparative anatomic effects of high-flow and low-flow phacoemulsification cataract surgery, future, updated systematic reviews integrating large-scale head-to-head comparisons are suggested.

This present study summarized the current evidence from RCTs, comparing anatomic effects between high-flow and low-flow phacoemulsification cataract surgery. However, we must acknowledge several limitations to the present study. First, CoE for each study outcome was judged suboptimal, and therefore we should interpret the study findings carefully. Also, we only evaluated the anatomic effects of the phacoemulsification cataract surgery, while future studies should investigate if these findings could be translated into functional outcomes, such as best-corrected visual acuity. Moreover, the present study only included participants who received phacoemulsification surgery for senile cataract from RCTs. Whether our findings could be applied to those receiving phacoemulsification surgery for solely refractive purposes, namely clear lens exchange, should be further investigated. Finally, our included RCTs mostly used the gravity-based infusion system, instead of newer technology, such as active fluidic system [e.g., Centurion system (Alcon)^®^], for phacoemulsification cataract surgery.

In conclusion, the low to very low CoE from the meta-analysis of the RCTs notwithstanding, high-flow phacoemulsification cataract surgery results in greater increase in postoperative CMT, but shows no difference in postoperative ECL and CCT, compared to low-flow phacoemulsification cataract surgery. Updated systematic reviews integrating future, large-scale, head-to-head comparisons of anatomic outcomes between these two phacoemulsification cataract surgery types are suggested.

## Data availability statement

The original contributions presented in the study are included in the article/Supplementary material, further inquiries can be directed to the corresponding authors.

## Author contributions

Conception, design, and data collection: P-CK and J-HH. Data analysis and interpretation: P-CK, J-HH, and S-CS. Preparation of the manuscript: P-CK, J-HH, Y-CS, and S-CS. Obtained funding: J-HH. All authors read and approved the final manuscript.

## Funding

This study was supported by grants from National Cheng Kung University Hospital, Tainan, Taiwan (NCKUH-11102004) to J-HH, and from the Ministry of Science and Technology (MOST 110-2314-B-006-086-MY3) to J-HH.

## Conflict of interest

The authors declare that the research was conducted in the absence of any commercial or financial relationships that could be construed as a potential conflict of interest.

## Publisher's note

All claims expressed in this article are solely those of the authors and do not necessarily represent those of their affiliated organizations, or those of the publisher, the editors and the reviewers. Any product that may be evaluated in this article, or claim that may be made by its manufacturer, is not guaranteed or endorsed by the publisher.
