# Supplementary material for: Comparative anatomical outcomes of high-flow vs. low-flow phacoemulsification cataract surgery: A systematic review and meta-analysis
Source: Front Med (Lausanne). 2022 Sep 28;9:1021941. doi: 10.3389/fmed.2022.1021941 (PMC9554630; doi:10.3389/fmed.2022.1021941)
Supplement: Supplementary file 1 [file Data_Sheet_1.PDF]

Supplementary data

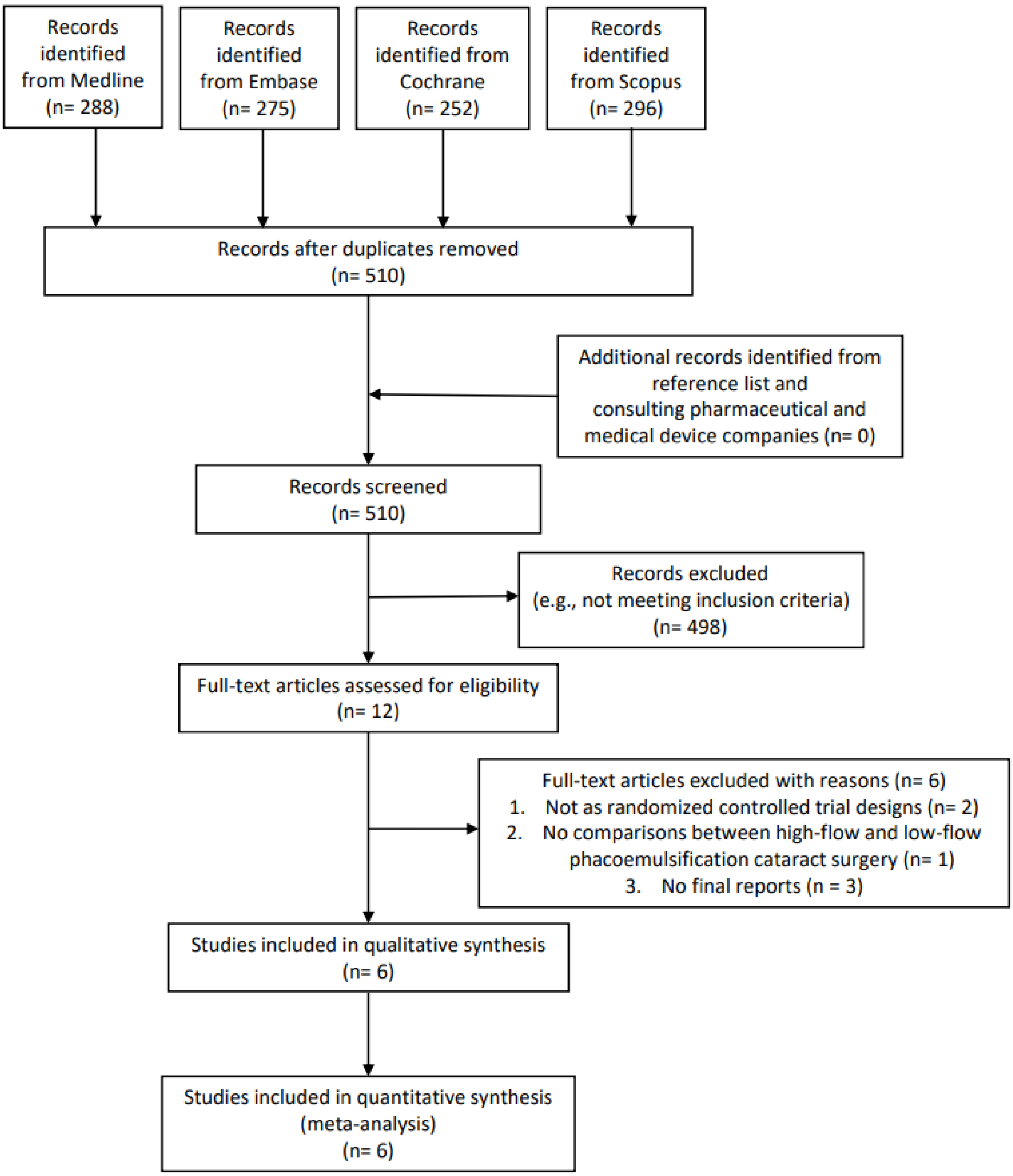

Figure S1. PRISMA flow diagram of study selection

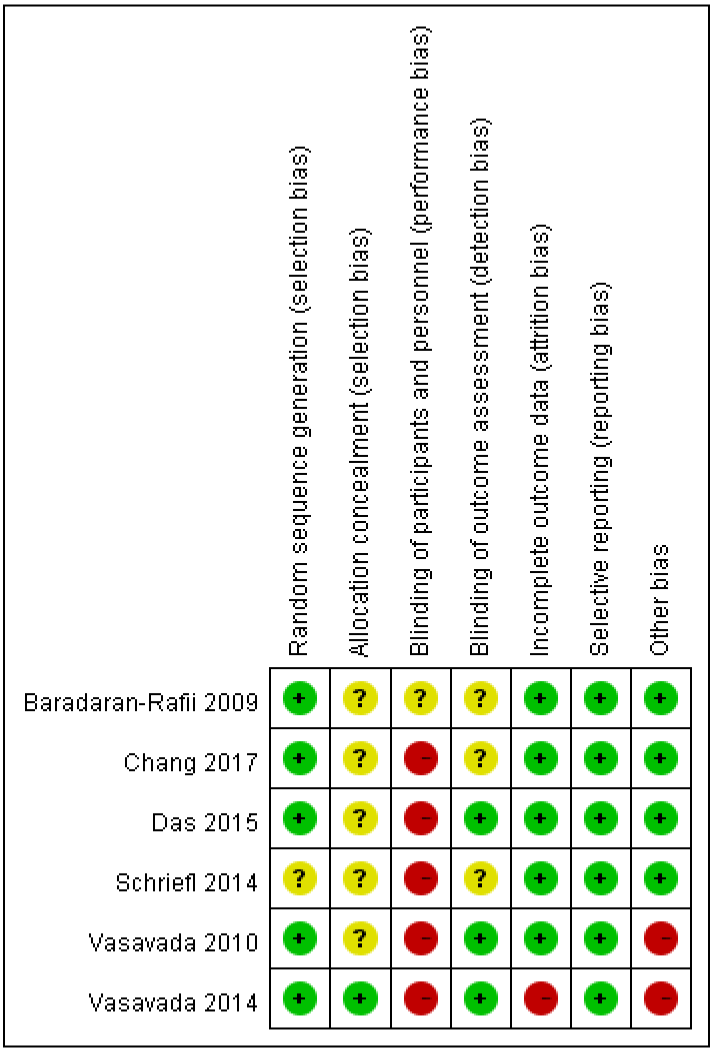

Figure S2. Risk of bias assessment of included studies

Table S1. Search strategy of the systematic review			
Database	#	Search syntax	Citations found
**Embase**	1	phacoemulsification/exp	17,049
	2	("len* emulsif*" OR phaco* OR phako* OR phacectom* OR phakectom*):ti,ab,kw,de	23,571
	3	'fluidics'/exp OR 'suction'/exp OR 'aspiration'/exp OR 'hydrodynamics'/exp OR 'flow'/exp OR 'parameters'/de OR 'physical parameters'/de	811,022
	4	(fluidic* OR flow* OR aspirat* OR suction* OR setting* OR hydrodynamic* OR dynamic* OR parameter* OR "cc/min*" OR "ml/min*"):ti,ab,kw,de	5,672,278
	5	'endothelium'/de OR 'cornea endothelium'/exp OR 'corneal endothelial cell loss'/exp OR 'endothelial cell'/exp	250,177
	6	(endothel* OR ECD):ti,ab,kw,de	629,516
	7	(#1 OR #2) AND (#3 OR #4) AND (#5 OR #6) AND [embase]/lim	623
	8	#7 AND ("randomized controlled trial"/de or "controlled clinical trial"/de or "randomization"/de or "intermethod comparison"/de or "double blind procedure"/de or "human experiment"/de OR (random* or placebo or assigned or allocated or volunteer or volunteers or (open NEXT/1 label) or ((double or single or doubly or singly) NEXT/1 (blind or blinded or blindly)) or "parallel group?" or crossover or "cross over" or ((assign* or match or matched or allocation) NEAR/5 (alternate or group? or intervention? or patient? or subject? or participant?)) OR (controlled NEAR/7 (study or design or trial))):ti,ab OR (compare or compared or comparison or trial):ti OR ((evaluated or evaluate or evaluating or assessed or assess) and (compare or compared or comparing or comparison)):ab) NOT (((random* NEXT/1 sampl* NEAR/7 ("cross section*" or questionnaire? or survey* or database?)):ti,ab not ("comparative study"/de or "controlled study"/de or "randomi?ed controlled":ti,ab or "randomly assigned":ti,ab)) OR ("Cross-sectional study"/de not ("randomized controlled trial"/de or "controlled clinical study"/de or "controlled study"/de or randomi?ed controlled:ti,ab or "control group?":ti,ab)) OR ((((case NEXT/1 control*) and random*) not randomi?ed controlled):ti,ab) OR ("Systematic review" not (trial or study)):ti OR (nonrandom* not random*):ti,ab OR "Random field*":ti,ab OR ("random cluster" NEAR/3 sampl*):ti,ab OR ((review:ab and review/it) not trial:ti) OR ("we searched":ab and (review:ti or review/it)) OR "update review":ab OR (databases NEAR/4 searched):ab OR ((rat or rats or mouse or mice or swine or porcine or murine or sheep or lambs or pigs or piglets or rabbit or rabbits or cat or cats or dog or dogs or cattle or bovine or monkey or monkeys or trout or marmoset?):ti and "animal	275

		experiment"/de) OR ("animal experiment"/de not ("human experiment"/de or "human"/de)))<br><br>Filter Source: Box 3.e, Technical Supplement to Chapter 4: Searching for and Selecting Studies. Cochrane Handbook for Systematic Reviews of Interventions Version 6. (Syntax Translated from Ovid Embase to Elsevier Embase.com.)	
**MEDLINE (Ovid)**	1	exp "phacoemulsification"/	11,249
	2	("len* emulsif*" OR phaco* OR phako* OR phacectom* OR phakectom*).mp	17,636
	3	exp "suction"/ OR exp "hydrodynamics"/	21,925
	4	(fluidic* OR flow* OR aspirat* OR suction* OR setting* OR hydrodynamic* OR dynamic* OR parameter* OR "cc/min*" OR "ml/min*").mp	3,875,560
	5	"endothelium"/ OR exp "endothelium, corneal"/ OR exp "corneal endothelial cell loss"/ OR exp "endothelial cells"/	99,147
	6	(endothel* OR ECD).mp	453,922
	7	(1 OR 2) AND (3 OR 4) AND (5 OR 6)	525
	8	7 AND (randomized controlled trial.pt. or controlled clinical trial.pt. or randomized.ab. or randomised.ab. or placebo.ab. or drug therapy.fs. or randomly.ab. or trial.ab. or groups.ab. not (exp animals/ not humans.sh.))	288
**Cochrane CENTRAL**	1	[mh "phacoemulsification"]	1494
	2	("len* emulsif*" OR phaco* OR phako* OR phacectom* OR phakectom*):ti,ab,kw	3481
	3	[mh "suction"] OR [mh "hydrodynamics"]	969
	4	(fluidic* OR flow* OR aspirat* OR suction* OR setting* OR hydrodynamic* OR dynamic* OR parameter* OR "cc/min*" OR "ml/min*"):ti,ab,kw	312,443
	5	[mh ^"endothelium"] OR [mh "endothelium, corneal"] OR [mh "corneal endothelial cell loss"] OR [mh "endothelial cells"]	1045
	6	(endothel* OR ECD):ti,ab,kw	22,094
	7	(#1 OR #2) AND (#3 OR #4) AND (#5 OR #6)	254
	8	#7 (Limits: in Cochrane Reviews, Cochrane Protocols, Trials)	252
**Scopus**	1	TITLE-ABS ("len* emulsif*" OR phaco* OR phako* OR phacectom* OR phakectom*) OR AUTHKEY ("len* emulsif*" OR phaco* OR phako* OR phacectom* OR	18,417

		phakectom*)	
	2	TITLE-ABS (fluidic* OR flow* OR aspirat* OR suction* OR setting* OR hydrodynamic* OR dynamic* OR parameter* OR "cc/min*" OR "ml/min*") OR AUTHKEY (fluidic* OR flow* OR aspirat* OR suction* OR setting* OR hydrodynamic* OR dynamic* OR parameter* OR "cc/min*" OR "ml/min*")	13,211,124
	3	TITLE-ABS (endothel* OR ECD) OR AUTHKEY (endothel* OR ECD)	467,952
	4	(INDEXTERMS ("clinical trials" OR "clinical trials as a topic" OR "randomized controlled trial" OR "Randomized Controlled Trials as Topic" OR "controlled clinical trial" OR "Controlled Clinical Trials" OR "random allocation" OR "Double-Blind Method" OR "Single-Blind Method" OR "Cross-Over Studies" OR "Placebos" OR "multicenter study" OR "double blind procedure" OR "single blind procedure" OR "crossover procedure" OR "clinical trial" OR "controlled study" OR "randomization" OR "placebo")) OR (TITLE-ABS-KEY (("clinical trials" OR "clinical trials as a topic" OR "randomized controlled trial" OR "Randomized Controlled Trials as Topic" OR "controlled clinical trial" OR "Controlled Clinical Trials as Topic" OR "random allocation" OR "randomly allocated" OR "allocated randomly" OR "Double-Blind Method" OR "Single-Blind Method" OR "Cross-Over Studies" OR "Placebos" OR "cross-over trial" OR "single blind" OR "double blind" OR "factorial design" OR "factorial trial"))) OR (TITLE (clinical trial OR trial OR rct* OR random* OR blind*))	
	5	#1 AND #2 AND #3 AND #4	296

Table S2. Risk-of-bias assessment of included trials		
Bias	Authors' judgement	Support for judgement
**Baradaran-Rafii 2009**		
Random sequence generation (Selection bias)	Low risk	"Patients were randomly assigned to 1 of 2 groups by permuted-block randomization with a block length of 4."
Allocation concealment (Selection bias)	Unclear risk	Allocation concealment was not described
Blinding of participants and personnel (Performance bias)	Unclear risk	Blinding of patients and personnel was not described.
Blinding of outcome assessment (Detection bias)	Unclear risk	Blinding of outcome assessors was not described.
Incomplete outcome data (Attrition bias)	Low risk	No missing outcome data was noticed in the study.
Selective reporting (Reporting bias)	Low risk	All outcomes were reported.
Other bias	Low risk	No other bias was detected.
**Chang 2017**		
Random sequence generation (Selection bias)	Low risk	"..., they received a number generated by Microsoft Excel computer software that randomized them to one of the 2 groups."
Allocation concealment (Selection bias)	Unclear risk	Allocation concealment was not described.
Blinding of participants and personnel (Performance bias)	High risk	"The patients were blinded during the course of the study, but the surgeon was not."
Blinding of outcome assessment (Detection bias)	Unclear risk	Blinding of outcome assessors was not described.
Incomplete outcome data (Attrition bias)	Low risk	Missing outcome data was clearly explained.
Selective reporting (Reporting bias)	Low risk	All outcomes planned in study protocol were reported.
Other bias	Low risk	No other bias was detected.

**Das 2015**		
Random sequence generation (Selection bias)	Low risk	"Patients recruited were randomized to either the high flow or the low flow group using a computerized random number table method, ..."
Allocation concealment (Selection bias)	Unclear risk	Allocation concealment was not described.
Blinding of participants and personnel (Performance bias)	High risk	"...and the study was patient and examiner masked." "The surgeon was not masked to the parameters used."
Blinding of outcome assessment (Detection bias)	Low risk	"...and the study was patient and examiner masked."
Incomplete outcome data (Attrition bias)	Low risk	No missing outcome data was noticed in the study.
Selective reporting (Reporting bias)	Low risk	Both primary and secondary outcomes were reported.
Other bias	Low risk	No other bias was detected.
**Schriefl 2014**		
Random sequence generation (Selection bias)	Unclear risk	"One of each patient's eyes was randomly assigned to group I, and the other eye was assigned to group II." No further description of the process.
Allocation concealment (Selection bias)	Unclear risk	Allocation concealment was not described.
Blinding of participants and personnel (Performance bias)	High risk	"In this prospective, randomized, single-blinded study,..." "... prospective, investigator blinded, ..." Patients and surgeons were probably not blinded.
Blinding of outcome assessment (Detection bias)	Unclear risk	Blinding of outcome assessors was not described.
Incomplete outcome data (Attrition bias)	Low risk	No missing outcome data was noticed in the study.
Selective reporting (Reporting bias)	Low risk	All outcomes were reported.
Other bias	Low risk	No other bias was detected.
**Vasavada 2010**		
Random sequence generation (Selection bias)	Low risk	"Randomization was by an opaque envelope system in which sequenced and sealed opaque envelopes containing 1 of the 2 options were prepared..."

Allocation concealment (Selection bias)	Unclear risk	Allocation concealment was not described.
Blinding of participants and personnel (Performance bias)	High risk	"In this prospective randomized patient- and examiner-masked study, ..." "The surgeon was not masked to the fluidic parameters."
Blinding of outcome assessment (Detection bias)	Low risk	"The same examiner performed all postoperative evaluations and was masked to the parameters used."
Incomplete outcome data (Attrition bias)	Low risk	No missing outcome data was noticed in the study.
Selective reporting (Reporting bias)	Low risk	All outcomes were reported.
Other bias	High risk	In Table 3 (preoperative and postoperative CCT and change over time), the study showed exactly the same result as another study (Vasavada 2014).
**Vasavada 2014**		
Random sequence generation (Selection bias)	Low risk	"Randomization was performed using a computer-generated random number table."
Allocation concealment (Selection bias)	Low risk	"Immediately prior to commencement of surgery, an unscrubbed nurse in the operating room informed the surgeon of the parameters to be employed for that particular patient."
Blinding of participants and personnel (Performance bias)	High risk	"No attempt was made to mask the surgeon to the surgical parameters being used." It was not described if the patients were blinded.
Blinding of outcome assessment (Detection bias)	Low risk	"A single examiner who was masked to the parameters used evaluated all of the postoperative observations."
Incomplete outcome data (Attrition bias)	High risk	In Table 4 (comparison of the incidence of corneal clarity, corneal edema, and Descemet's membrane folds), only 30 patients were included in the high parameters group, while the missing ones were not explained.
Selective reporting (Reporting bias)	Low risk	All outcomes were reported.
Other bias	High risk	In Table B (comparison of preoperative and postoperative central corneal thickness), the study showed exactly the same result as another study (Vasavada 2010).
